# Emergent laparoscopic surgical intervention for perforated hemorrhagic cholecystitis with hemodynamic instability

**DOI:** 10.1093/jscr/rjac454

**Published:** 2022-10-19

**Authors:** Alexander S Baier, Dorothy Liu, Jonson Yee, Nicole Cherng, Hongyi Cui, Edward Kim

**Affiliations:** Program in Molecular Medicine, University of Massachusetts Chan Medical School, Worcester, MA, USA; Department of Surgery, University of Massachusetts Memorial Medical Center, Worcester, MA, USA; Department of Surgery, University of Massachusetts Memorial Medical Center, Worcester, MA, USA; Department of Surgery, University of Massachusetts Memorial Medical Center, Worcester, MA, USA; Department of Surgery, University of Massachusetts Memorial Medical Center, Worcester, MA, USA; Department of Surgery, University of Massachusetts Memorial Medical Center, Worcester, MA, USA

## Abstract

Hemorrhagic cholecystitis is a rare diagnosis that closely mimics acute cholecystitis. Physical examination, laboratory studies and, in particular, computed tomography imaging allow for rapid diagnosis, stabilization and emergent surgical intervention. We describe our experience with three patients requiring emergent surgical intervention for hemorrhagic cholecystitis with unique clinical features including decreased platelet function due to liver cirrhosis, dual antiplatelet therapy and intraoperative finding of cholecystohepatic communication. Furthermore, we provide video recordings of two cases highlighting the severity of the disease. All presented patients were hemodynamically unstable and showed peritoneal signs on exam. Laboratory studies revealed moderate anemia and leukocytosis, while computed tomography suggested hemorrhage in the gallbladder. All patients required blood transfusions during their care and underwent laparoscopic cholecystectomy. Hemoperitoneum and gallbladder perforation were confirmed intraoperatively. Patients fully recovered without significant postoperative complications due to expedited operative management.

## INTRODUCTION

Hemorrhagic cholecystitis (HC) is a rare and potentially fatal cause of acute cholecystitis where rapid diagnosis and operative management to remove the gallbladder and control intraperitoneal hemorrhage are critical for a positive outcome [1]. Diagnosis of HC by clinical presentation is challenging, as symptoms such as right upper quadrant pain, fever and leukocytosis are often indistinguishable from acute cholecystitis [2]. Effective management of HC requires prompt resuscitation and urgent surgical intervention. This contrasts with the literature examining the relative benefits of ‘early cholecystectomy’ versus ‘late cholecystectomy’ in acute cholecystitis, where early intervention is usually defined as occurring within 72 hours of patient presentation [3]. Herein, we describe our experience with three operative cases of HC.

## CASE 1

**Figure 1:**
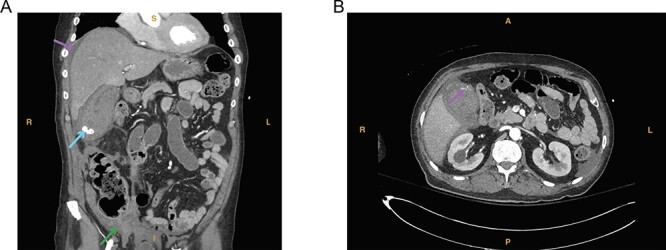
(**A**) Computed tomography imaging from Case 1 showing a distended gallbladder with multiple calculi (cyan arrow) as well as hyperdense intraluminal fluid (magenta arrow) with collection in the pelvis (green arrow) consistent with hemorrhage. Present in the figure, the letters S, L, I and R denote superior, left, inferior and right, respectively. (B) Computed tomography imaging from Case 1 showing the tortuous path of a cholecystic artery branch (magenta arrow) at the gallbladder fundus. Present in the figure, the letters A, L, P and R denote anterior, left, posterior and right, respectively.

A 74-year-old man with a past medical history of type II diabetes mellitus, recent COVID-19 infection with prolonged hospital course and ventilator dependence requiring tracheostomy status post decannulation, coronary artery disease and myocardial infarction status post three-vessel coronary artery bypass graft in 1991 and drug-eluting stent placement in 2020 on dual antiplatelet therapy (DAPT; aspirin and clopidogrel) presented with acute onset epigastric and right upper quadrant pain that started 3 hours prior to presentation from a rehabilitation facility. In the emergency department, the patient was hypotensive (systolic blood pressure of 70 mmHg) with a transient response to crystalloid resuscitation. Laboratory studies revealed a hemoglobin of 8.9 g/dl, a hematocrit of 27%, leukocytosis (21.6 × 103/μl, normal range 4.3–10.8 × 103/μl), slightly elevated lactic acid to 1.9 mmol/L (normal range 0.3–1.9 mmol/l) and elevated creatinine serum to 1.43 mg/dl. Computed tomography of the abdomen and pelvis revealed a distended gallbladder containing multiple calculi and hyperdense intraluminal content (Fig. 1A). A prominent tortuous cholecystic artery branch was noted at the fundus (Fig. 1B). Hemoperitoneum with concern for acute hemorrhage was suspected, given fluid collection in the pericholecystic, perihepatic and perisplenic spaces.

On exam, the patient was visibly uncomfortable complaining of generalized abdominal and back pain with a peritoneal sign. Given his concerning abdominal exam, hemodynamic instability and radiographic image suggestive of a hemorrhagic process likely involving the gallbladder, the decision was made for emergent operative intervention.

**Figure 2:**
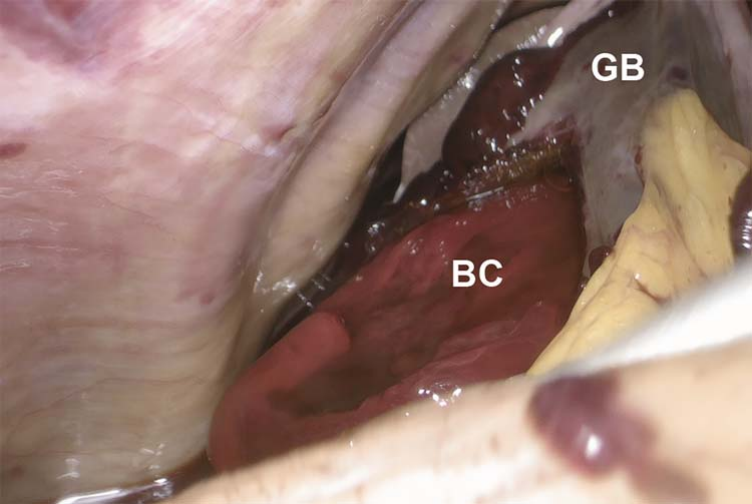
Intraoperative laparoscopic image showing the protrusion of a bright red clot (BC) from a perforation of the gallbladder (GB) wall upon cephalad retraction of the gallbladder.

We started the case with diagnostic laparoscopy. Entry into the abdomen revealed approximately 500 cc of blood clot and hemorrhagic fluid. There were some omental adhesions to the anterior abdominal wall which we dissected sharply. Upon cephalad retraction of the gallbladder, obvious perforation of the gallbladder wall with a large protruding clot was appreciated (Fig. 2). Intraoperative hematocrit trended down to 18%, and the patient received 2 units of packed red blood cells and 1 unit of platelets. With meticulous dissection, critical view and hemostasis were achieved. Large clots were evacuated using an EndoCatch™ bag (Medtronic, Minneapolis, MN). After copious irrigation, we did not identify another source of hemorrhage. A 24-French Blake drain was left in the gallbladder fossa. Patient was transferred to the surgical intensive care unit (SICU) for postoperative monitoring (Supplementary Video 1).

The patient required an additional unit of packed red blood cells on postoperative day 1 and again on postoperative day 2 in the setting of decreased hematocrit. DAPT therapy was restarted on postoperative day 2. On postoperative day 6, the patient was discharged in stable condition to an inpatient rehabilitation facility. Surgical pathology demonstrated marked edema, transmural acute inflammation with serositis and mucosal ulceration. Focal mucosal glandular proliferation suggestive of pyloric gland adenoma without any evidence of malignancy was incidentally noted.

## CASE 2

**Figure 3:**
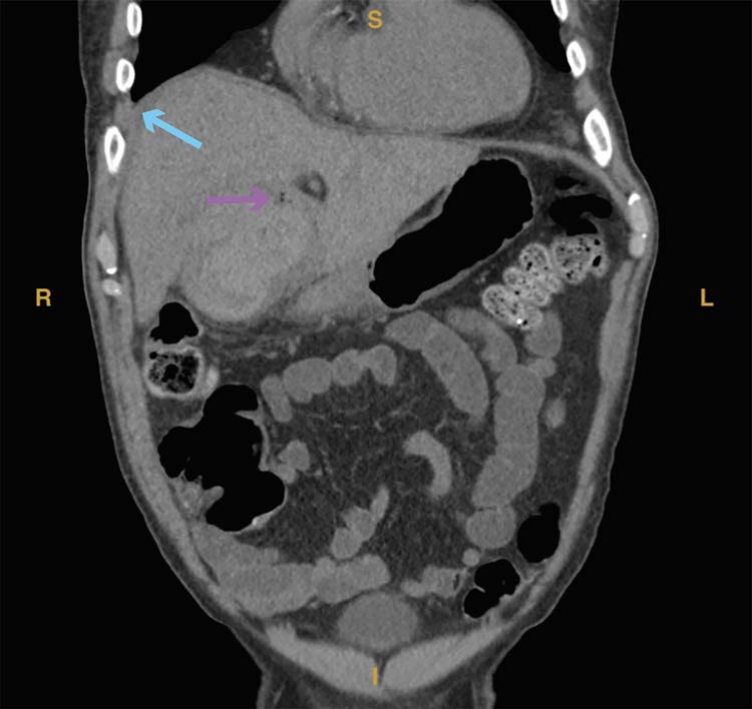
Computed tomography imaging from Case 2 showing air in the gallbladder wall (magenta arrow) as well as intraluminal fluid (cyan arrow) consistent with hemorrhage. Present in the figure, the letters S, L, I and R denote superior, left, inferior and right, respectively.

A 69-year-old man with a past medical history of hypertension, hyperlipidemia and CVA status post tPA in 2020 with no residual defects presented with 5 days of right upper quadrant pain and 1 day of nausea, bloody diarrhea and anorexia. In the emergency department, the patient was hemodynamically unstable, with a systolic blood pressure of 80 mmHg, for which he received 2 l of crystalloid bolus. Laboratory studies revealed a hemoglobin of 9.6 g/dl, a hematocrit of 28%, an elevated serum creatinine of 3.09 mg/dl (normal range 0.9–1.3 mg/dl), leukocytosis (29 × 103/μl, normal range 4.3–10.8 × 103/μl) and hyponatremia (129 mMol/l, normal range 135–145 mMol/l). Computed tomography showed acute acalculous HC in addition to air in the gallbladder wall, possibly due to gangrene or fistula (Fig. 3).

On exam, the patient was focally peritonitic to the right upper quadrant with radiation to the epigastric region. In the setting of hemodynamic instability, the radiographic findings and peritoneal sign, the decision was made for emergent operative intervention.

**Figure 4:**
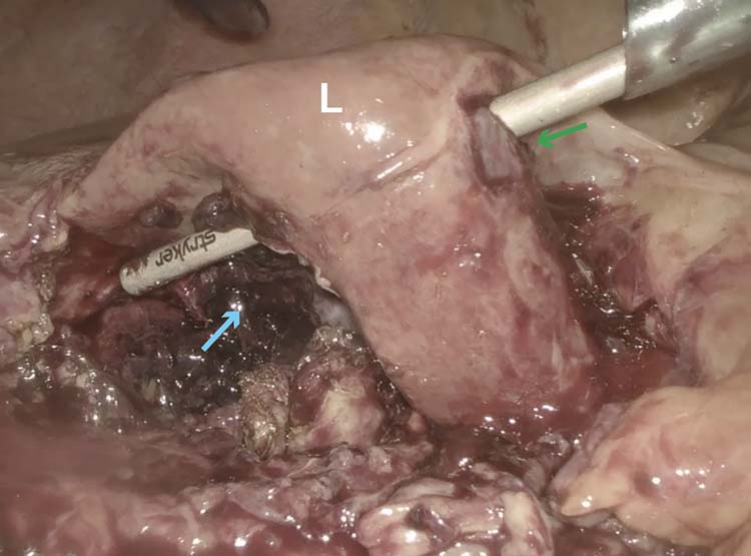
Intraoperative laparoscopic image showing obliteration of segment 5 (cyan arrow) of the liver (L) with perforation into segment 4B (green arrow).

We proceeded with a laparoscopic approach, and entry into the abdomen revealed hemoperitoneum with active bleeding around the liver. The omentum was adherent to the presumed location of the gallbladder along with section 4B of the liver and along the right side of the falciform ligament. Blunt dissection to sweep down the omentum revealed a large cavity in segment 5 of the liver filled with necrotic tissue and blood clots with perforation into segment 4B (Fig. 4). This cavity appeared to be in communication with the posterior wall of the gallbladder, and it was clear the lateral wall of the gallbladder was also compromised. Out of concern for ongoing hemorrhage, the patient was given 2 units of packed red blood cells intraoperatively. We separated the gallbladder from the liver bed where it had not already perforated into the liver using top-down dissection to the level of the infundibulum, but due to inflammation we were unable to visualize the cystic artery or duct. The decision was made to transect the gallbladder as low as possible, and we were able to visualize both the anterior and posterior walls of the gallbladder ensuring adequate closure of the lumen. Two 0-PDS Endoloop ligatures (Ethicon Inc., Somerville, NJ) were placed around the infundibulum. We removed the gallbladder along with large amounts of necrotic tissue from the liver, described by pathology as inflamed parenchymal tissue and blood clot without evidence of malignancy, using an EndoCatch™ bag (Medtronic, Minneapolis, MN), and active bleeding in the cavity was addressed with Surgicel Nu-Knit gauze (Ethicon Inc., Somerville, NJ). A 19-French round Blake drain was left in the gallbladder fossa, and the patient was transferred to the SICU for postoperative monitoring (Supplementary Video 2).

The patient was transferred to a non-intensive care unit on postoperative day 2 and required an esophagogastroduodenoscopy for a hemodynamically significant gastrointestinal bleed revealing an actively bleeding gastric ulcer resolved with epinephrine injection and four clips on postoperative day 5. On postoperative day 6, the patient was found to have atrial fibrillation with new onset rapid ventricular response, rate controlled with metoprolol. The patient was discharged to home in stable condition on postoperative day 8.

## CASE 3

**Figure 5:**
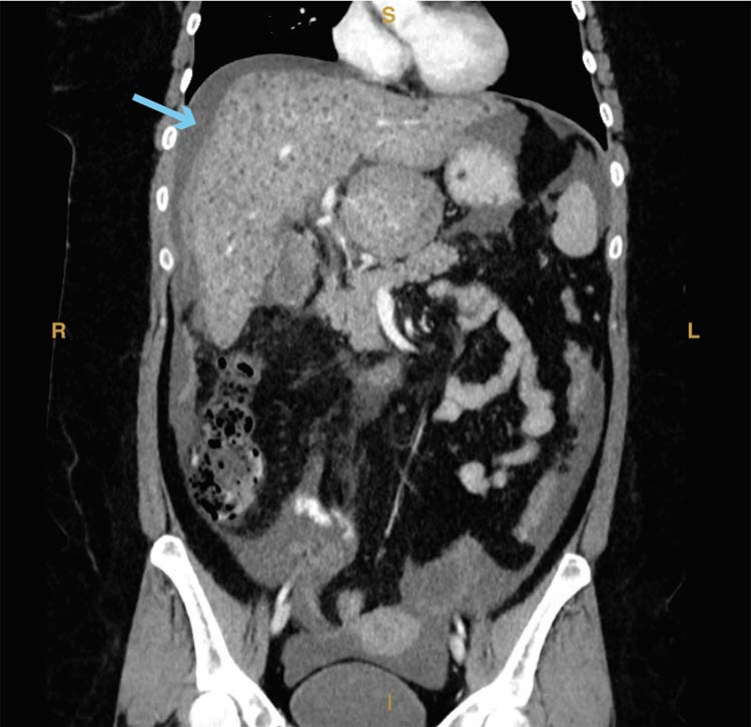
Computed tomography imaging from Case 3 showing significant hemoperitoneum (cyan arrow). Present in the figure, the letters S, L, I and R denote superior, left, inferior and right respectively.

A 23-year-old woman with a past medical history of autoimmune hepatitis with ascites on azathioprine and prednisone presented from an outside hospital with 24-hour acute onset of worsening right upper quadrant pain and mild nausea. Patient’s model for end-stage liver disease score based on an INR value from 3 months prior is 14. Prior to being transferred to our emergency department, the patient was hypotensive (systolic blood pressure of 90 mm Hg) and given crystalloid resuscitation. Initial workup revealed a hemoglobin of 8.9 g/dl, a hematocrit of 25.8%, elevated lactic acid to 2.7 mmol/l (normal range 0.3–1.9 mmol/l) and elevated total bilirubin of 2.4 mg/dl (normal range 0.3–1.2 mg/dl). Computed tomography of the abdomen and pelvis revealed significant hemoperitoneum with suspected gallbladder perforation, as well as small calculi in the gallbladder neck (Fig. 5).

On exam, the patient was in mild discomfort with some pain localized to the right upper quadrant without rebound or guarding. However, in the setting hemodynamic instability for which the patient received 2 units of packed red blood cells in the emergency department, downtrending hemoglobin and radiographic findings of hemoperitoneum, the decision was made for urgent surgical intervention despite poor liver reserve due to patient’s Child-Pugh class B cirrhosis.

We started with a diagnostic laparoscopy, and during induction, the patient received one unit of fresh frozen plasma. Entry into the abdomen revealed approximately 1500 cc of non-clotting blood, present in all four quadrants. The liver was severely cirrhotic, and inspection of the gallbladder revealed a large perforation running from the fundus to the level of the infundibulum. Using blunt and sharp dissection, the cystic artery and duct were visualized, clipped and the stumps further ligated with 2 0-PDS Endoloop ligatures (Ethicon Inc., Somerville, NJ). The gallbladder was retrieved in a specimen bag and active bleeding in the fossa was controlled with Surgicel Nu-Knit gauze (Ethicon Inc., Somerville, NJ) secured with a 2-0 Vicryl stitch and two surgical clips. After aspiration of the hemoperitoneum, a 19-French JP drain was placed in the subhepatic space and gallbladder fossa. The patient tolerated the procedure well and was transferred to the SICU for postoperative monitoring.

The patient received an additional 2 units of fresh frozen plasma postoperatively and an additional unit of packed red blood cells on postoperative day 1. The patient remained hemodynamically stable thereafter and was transferred to a non-intensive care unit on postoperative day 3. The patient was discharged without complication on postoperative day 5.

## DISCUSSION

Notably in our first case, the patient’s condition occurred in the setting of recent COVID-19 infection and DAPT. A correlation between viral illness and acute cholecystitis is established [4], and there have been multiple case reports that have drawn an association between COVID-19 infection and acute cholecystitis [5, 6]. We believe this calls for raising the index of suspicion for cholecystitis in patients with ongoing or recent COVID-19 infection. Additionally, extensive review of the literature revealed we are the first to report HC in a patient on DAPT. Our patient’s PRECISE-DAPT [7] score was 30 two weeks prior to admission. This placed him at high risk for a major bleeding event and his score had increased to 39 at the time of admission. For patients on DAPT, a high PRECISE-DAPT score in the setting of acute onset epigastric or right upper quadrant should provide a higher index of suspicion for HC.

Our second case also presented with unique features, namely our mid-operative discovery that the gallbladder had perforated into the liver, causing parenchymal necrosis obliterating segment 5 with intrahepatic perforation into segment 4b. The etiology of this patient’s HC and complications is unclear but may be due to vascular disease causing compromise of the gallbladder wall. Cholecystohepatic communication is a rare complication and has never been reported in a case of HC [8]. Thus, no standard management exists for this complication; in our patient, we evacuated the necrotic tissue and prescribed a course of cefpodoxime for broad spectrum antibacterial coverage.

Our third case reinforced the efficacy of prompt hemodynamic resuscitation and laparoscopic cholecystectomy in patients with suspected HC. This patient had decreased synthetic liver function, which is both a risk factor for surgical complications and may have contributed to the development of HC through impaired clotting. However, laparoscopic techniques can reduce the risk to cirrhotic patients [9] and our patient made a full recovery without complications.

Despite the demonstrated variation in presentation, management of HC should be broadly focused on prompt diagnosis followed by emergent surgical intervention. Factors contributing to HC include bleeding risks such as anticoagulation or cirrhosis, blunt trauma, peripheral neuropathies which can mask symptoms, and vascular disease which can compromise the gallbladder wall [10]. Symptoms of acute cholecystitis paired with physical findings of hemodynamic instability, or radiographic evidence of hemoperitoneum should give clinicians a high index of suspicion for HC, with computed tomography being a particularly valuable imaging modality. In preparation for surgery, a type and screen should be performed, and blood products ordered as a precaution, as all three patients required transfusions. While we started our cases with a diagnostic laparoscopy, we recognize open surgery or conversion may be preferable in cases with a difficult abdomen due to prior surgery or hemodynamic instability non-responsive to resuscitation. We hope that this case series, with its various unique clinical features further complicating a rare diagnosis, will enable our peers to more timely recognize and respond to this potentially fatal condition. Additionally, all patients made a full recovery without significant follow-up events, demonstrating surgical management of HC can be undertaken laparoscopically while preserving patient safety.

## Supplementary Material

HC_case1_tbac022

HC_case2_tbac022
